# How registry data can improve outcomes from joint replacement – a seminal paper

**DOI:** 10.1080/17453674.2020.1763567

**Published:** 2020-05-12

**Authors:** Keith Tucker

**Affiliations:** Norwich England, Chairman of ODEP and the Beyond Compliance Advisory Group, United Kingdom

Charnley, McKee and Ling, arguably the pioneers of Total Hip Replacement (THR), all recommended that there should be some sort of register for hip replacements. Perceptively, Sir John wrote in 1972: *“Serious consideration should be given to establishing a Central Register to keep a finger on the pulse of total implant surgery on a nation-wide basis.”* However, it was Peter Herberts and Lennart Ahnfelt, following the example set by the Swedish Knee Arthroplasty Register (SKAR 1975), who set up the world’s first hip register in 1979, the Swedish Hip Arthroplasty Registry (SHAR).

Nowadays we all realise how registers have formed the basis for the multitude of observational studies that have been conducted over the past 40+ years. What we have also, increasingly realized, is that collecting data is only half the job. It is what can be learnt from the data that is so important.

In their article: *“Long-term registration has improved the quality of hip replacement”* Herberts and Malchau (2000) reviewed the Swedish THR Register comparing the 160,000 cases contained within it. Not only did they show how huge amounts of observational data, can be collected, they introduced us to a methodology of its interrogation and ways of showing its value

They made it clear from the outset that it was their primary aim to make hip replacement safer and more reliable for patients. Another aim was to evaluate the performance of surgeons and implants whilst getting the information into the public domain, so as to influence health care policy. They argued that registry data, properly analysed, was more likely to give a true picture of the effectiveness of the operation than a clinical trial or RCT (Randomized Control Trial). Importantly, they argued that registry data will include the results from all the surgeons doing the operation and not just from those in the originating centre.

The tools they used are second nature to us now. Revision of the implant was the trigger for further analysis whether it be surgical performance, the type of implant, the use of cement (and type of cement) etc. The risks of infection and its control were drawn out of the database besides the other common indications for revision. They defined revision whilst accepting that there are other definitions than their own.

Anyone who wants to make a registry work properly, will know that the stakeholders and all the people who contribute data, must gain benefit from it. It is pointless having a registry without “feedback”. Herberts and Malchau included interesting and pertinent demographic data. They showed graphs on trends, just as we all do nowadays. Importantly, they showed how, with SHAR, the revision rate in Sweden dropped. Importantly for many of us, they compared their “Post Registry” revision rate with reported revision rates in other countries.

Over the past 20 years there has been much debate as to which statistical methodology is the best for registry observational data. They used KM (Kaplan–Meier) which set the benchmark and has hardly ever been superseded.

The hallmarks of a first-class registry, as defined by ISAR (The International Society of Arthroplasty Registries) are completeness (all hospitals taking part), compliance (all operations being up-loaded) and linkability (the ability to link primary to revision operations in the same patient). Herberts and Malchau pioneered the importance of these standards whilst commenting on the importance they attached to validation. Validation is another of the standards expected by ISAR to qualify a registry to be in the first division. It is often difficult, time consuming and expensive but an effective method is discussed in the text.

Cost would have inevitably been a question that any reader would have been thinking about whilst reading this article in 2000. The authors pointed out that the costs of revision operations that could be prevented by surgeons taking heed of the findings reported in the article, would far outweigh the costs of the register and the expense of data analysis. They were fortunate that the advent of the registry was well timed in terms of the IT revolution that we all witnessed, towards the end of the last century. It has made data collection much easier, more accurate and much easier to analyse.

There must have been thousands of surgeons who read this article when it was published in 2000 and who were immediately struck by its importance. Surely, it can be assumed that it would not just have been UK surgeons, who had learned from the article that their revision rate was probably 3 times the Swedish rate, that their country also needed a joint replacement registry. There can be no doubt that SHAR and this paper accelerated the introduction of many of the registries we have today.

What is even more impressive is that when one reads the article again, very nearly 20 years later we still use virtually the same standards and methodology and reporting methods in most of the world’s registries today.

Of course, it is not just hips nowadays but all joints. The basics has been copied by other subspecialities in orthopaedics and increasingly in other medical specialities.

At the time this paper was revolutionary. There are often some who will dismiss/ridicule a novel idea which doesn’t sit comfortably with their own beliefs. It helped their cause that both Herberts and Malchau have always been held in the highest regard in terms of honesty and diligence, throughout the world.

This article, which many will now regard as seminal, set the pattern for the reporting of registry data, the methodology that should be used and introduced many of the important definitions that we have been using ever since.

**Keith Tucker** *Norwich England* *Chairman of ODEP and* *the Beyond Compliance Advisory Group* *United Kingdom* *E-mail:* KTUCKER77@aol.com
